# Severe Prolonged Drought Favours Stress-Tolerant Microbes in Australian Drylands

**DOI:** 10.1007/s00248-023-02303-w

**Published:** 2023-10-25

**Authors:** Premchand Maisnam, Thomas C. Jeffries, Jerzy Szejgis, Dylan Bristol, Brajesh K. Singh, David J. Eldridge, Sebastian Horn, Jeff Chieppa, Uffe N. Nielsen

**Affiliations:** 1 Hawkesbury Institute of Environment, Western Sydney University, Penrith, NSW Australia; 2 School of Science, Western Sydney University, Penrith, NSW Australia; 3 Global Centre for Land Based Innovation, Western Sydney University, Penrith, NSW Australia; 4 Centre for Ecosystem Science, School of Biological, Earth and Environmental Sciences, University of New South Wales, Sydney, NSW Australia

**Keywords:** Drylands, Climate change, Rainfall manipulation, Drought, Soil bacterial and fungal communities

## Abstract

Drylands comprise one-third of Earth’s terrestrial surface area and support over two billion people. Most drylands are projected to experience altered rainfall regimes, including changes in total amounts and fewer but larger rainfall events interspersed by longer periods without rain. This transition will have ecosystem-wide impacts but the long-term effects on microbial communities remain poorly quantified. We assessed belowground effects of altered rainfall regimes (+ 65% and -65% relative to ambient) at six sites in arid and semi-arid Australia over a period of three years (2016–2019) coinciding with a significant natural drought event (2017–2019). Microbial communities differed significantly among semi-arid and arid sites and across years associated with variation in abiotic factors, such as pH and carbon content, along with rainfall. Rainfall treatments induced shifts in microbial community composition only at a subset of the sites (Milparinka and Quilpie). However, differential abundance analyses revealed that several taxa, including Acidobacteria, TM7, Gemmatimonadates and Chytridiomycota, were more abundant in the wettest year (2016) and that their relative abundance decreased in drier years. By contrast, the relative abundance of oligotrophic taxa such as Actinobacteria, Alpha-proteobacteria, Planctomycetes, and Ascomycota and Basidiomycota, increased during the prolonged drought. Interestingly, fungi were shown to be more sensitive to the prolonged drought and to rainfall treatment than bacteria with Basidiomycota mostly dominant in the reduced rainfall treatment. Moreover, correlation network analyses showed more positive associations among stress-tolerant dominant taxa following the drought (i.e., 2019 compared with 2016). Our result indicates that such stress-tolerant taxa play an important role in how whole communities respond to changes in aridity. Such knowledge provides a better understanding of microbial responses to predicted increases in rainfall variability and the impact on the functioning of semi-arid and arid ecosystems.

**Supplementary Information:**

The online version contains supplementary material available at 10.1007/s00248-023-02303-w.

## Introduction

Drylands are defined as regions with arid, semi-arid and dry sub-humid climates and comprise about one-third of Earth’s terrestrial surface area and support over 38% of the global human population [1]. Climate change is expected to expand this area by altering rainfall regimes globally [2]. Moreover, drylands are projected to experience significant changes in rainfall, including changes in total amounts but also fewer but larger rainfall events interspersed by longer periods without rain [3, 4]. Increased frequency of dry periods coupled with low nutrient availability will impose significant constraints on soil biota [5]. Soil microorganisms are responsible for essential belowground ecosystem processes, such as decomposition and mineralization of organic matter and nutrient cycling, which in turn affects the above plant community [6–8]. Changes in precipitation patterns or increasing aridity can cause progressive shifts in diversity, composition, and functional attributes of soil microbial communities [9, 10], often with consequences for ecosystem processes and plant community dynamics [11]. This has important implications for soil organic carbon (C) and nutrient cycling processes that are largely driven by rainfall in already water-limited dryland ecosystems [12, 13].

The predicted changes in rainfall can alter soil microbial communities through impacts on soil water availability but also indirectly through affecting plant community composition and soil physiochemical properties [14, 15]. For example, prolonged drought stress favours stress-tolerant plant species and their root-associated microorganisms, thus impacting the soil microbial community [16]. By contrast, large rainfall events or periods of above-average rainfall may induce plant growth and promote C and nutrient cycling [17]. While we have a broad understanding of climate change impacts on soil microbial communities, it is still unclear whether changes in microbial communities due to prolonged drought may have long term effects, and how stable microbial communities are in response to rainfall variability, especially in drylands [18]. Moreover, microbes are interconnected and form various ecological relationships [19], but the effects of climate change on microbial network complexity and stability remains uncertain.

As soils are dynamic systems, most soil microorganisms have adapted strategies to cope with changing environmental conditions [20]. However, soil microbial responses to altered precipitation vary widely among taxa and functional types [21]. For instance, oligotrophic soil microbes, such as the bacterial phyla Actinobacteria and Chloroflexi and the fungal phylum Ascomycota, are considered to be stress tolerant due to their thick cell walls and their ability to form spores and hyphal networks allowing them to adapt and thrive in harsh environments [11, 22]. Dryland soil microbes are known to be well-adapted to infrequent, intense, and unpredicted rainfall (Li et al., 2009); however, climate change is expected to introduce more extreme variation with unknown consequences on the stability and resilience of the soil microbiome [20]. In addition, climatic condition such as drought can be considered as two-part disturbances where drying and rewetting is always coupled and such disturbances can cause physiological and chemical perturbations to the soil system affecting microbial community [23]. These fluctuations in soil moisture also alter particle aggregate size, reshaping the soil structure and affecting microbial mobility and colonization [23]. The ability of microbes to acclimatise and adapt to novel rainfall regimes will depend on the degree of perturbation and their innate metabolic plasticity [11]. Furthermore, the predicted climate changes are expected to impact microbial ecological network structure [24], with bacterial interactions considered more sensitive to perturbations [16], Zheng et al*.*, 2021). Although several studies have reported relationships between network structure and dryland ecosystem functioning and stability [25, 26], little is known about whether and how the microbial networks change under future climate change scenarios and the potential cascading effects on functioning.

The effect of altered rainfall regimes on belowground assemblages has received increasing attention over the past couple of decades [17, 27]. Microbial community composition and diversity have been shown to be affected by experimentally altered precipitation regimes and show predictable shifts across precipitation gradients, mostly mediated by changes in plant community diversity and soil properties [28]. Studies with both increased and reduced rainfall treatments across multiple sites show significant changes in microbial communities where reduced rainfall increases the dominance of stress-tolerant microbes and vice versa in response to increased rainfall [27, 28], while other studies found no treatment effects [29, 30]. The seemingly idiosyncratic responses to altered precipitation may be due to several factors, such as variation in seasonal rainfall, the sensitivity of vegetation to altered rainfall, soil organic substrate availability, and differences in microbial community composition and functional attributes [31]. Comprehensive long-term rainfall manipulation studies that consider both the amount of precipitation (i.e., increased and decreased), as well as event size and frequency, seasonal shifts in rainfall patterns, varying soil nutrients, and microbial functional group responses to changing precipitation, are required to help address this knowledge gap [17, 20]. Addressing these shortcomings will improve our understanding of the roles of soil microbes in biogeochemical cycles in dryland ecosystems across spatial scales under climate change scenarios [32].

The present study aimed to understand the effects of altered precipitation regimes on the structure and stability of dryland soil microbial communities, and whether changes in the soil physiochemical properties over time have a significant effect on microbial community structure. To address this, we examined the temporal dynamics of soil microbial communities in a long-term *in situ* rainfall experiment in dryland ecosystems over four consecutive years (2016–2019). We assessed bacterial and fungal community responses to altered rainfall at six Australian dryland sites that vary in mean annual precipitation (MAP, ~ 200 to 450 mm year^−1^), with varying aridity where two were classified as arid and four as semi-arid. Rainfall manipulations were established in the spring of 2016 (~ 65% reduction and ~ 65% increase relative to ambient rainfall) but sites were co-incidentally also subjected to a natural prolonged drought (2017–2019). We used co-occurrence network analyses to investigate shifts in microbial community structure pre- and post-drought (2016 v 2019), contrasting effects at the two arid and the four semi-arid ecosystems. We hypothesised that bacterial and fungal communities would show differential responses to temporal changes in precipitation. Fungi were expected to be less sensitive to prolonged drought stress than bacteria because of their ability to accumulate osmoregulatory solutes and produce hyphae that can scavenge substrates even in very dry soils [22]. Additionally, we hypothesised that drought-induced resource limitation would result in a higher relative abundance of oligotrophic taxa such as Actinobacteria, Chloroflexi, and Ascomycota and that they will have an impact on overall microbial community assembly in semi-arid and arid ecosystems.

## Materials and Methods

### Experimental Design and Sampling

The study was conducted at rainfall manipulation facilities at six sites in south-eastern Australia established to assess the effect of altered rainfall regimes on ecosystem structure and functioning in dryland ecosystems. The six sites represent different rainfall regimes with a west-to-east increase in rainfall and greater interannual rainfall variability (CV) at the northern sites. The sites have different vegetation types (Supplementary Material 1, Table S1) and soil properties [33], different mean annual precipitation (MAP, ranging from 209 to 494 mm year ^−1^) and temperature (MAT, ranging from 18 to 21º C). The long-term aridity index (AI) ranges from 3.7 to 8.9 across the six sites (Table 1). The AI was calculated as the ratio of mean annual potential evapotranspiration and mean annual precipitation (i.e. higher values equal greater aridity), with climate data retrieved from SILO [34] using values from 1976 to 2005 as a reference period [33]. Of note, the two semi-arid sites in New South Wales (NSW), Cobar and Nyngan, are geographically close to each other, as are the two arid sites in NSW, Milparinka and Broken Hill. Similarly, the two semi-arid sites in Queensland (QLD), Quilpie and Charleville are also closely located. Rainfall manipulation facilities were placed in homogeneous grass and shrub-dominated areas at all sites to reduce variation associated with vegetation structure. Weather stations were established at each site in 2016 to monitor rainfall using a rain gauge (CS701, Campbell Scientific), temperature and relative humidity (Vaisala, HMP60) and photosynthetically active radiation (PAR) (Apogee, SQ-110). Additionally, soil moisture was recorded every 15 min using water content reflectometers (Campbell Scientific, CS616) in each plot at each site to verify treatment effects. Data were stored on a Campbell Scientific CR1000 control module powered by a solar panel and battery, with data transferred via mobile network.

**Table 1:** List of dryland field sites including site name, ecosystem type, latitude, longitude, and long-term mean annual precipitation (MAP, in mm year^−1^), inter-annual rainfall variability (Coefficient of Variation, CV), mean annual temperature (MAT, in ºC and aridity index (AI)). Long term mean annual rainfall and temperature based on WorldClim2 (1970–2000; [35])

Site name	Ecosystem type	Latitude	Longitude	MAP	CV	MAT	AI
Broken Hill	Arid shrubland	-32.0°	141.6°	258	0.28	18.4	6.1
Cobar	Semi-arid woodland	-31.8°	145.6°	363	0.27	18.3	4.4
Nyngan	Semi-arid shrubland	-31.7°	146.6°	405	0.31	18.5	3.7
Milparinka	Arid shrubland	-29.6°	141.7°	209	0.52	20.0	8.9
Quilpie	Semi-arid woodland	26.6°S	144.6°	377	0.57	21.5	5.5
Charleville	Semi-arid woodland	26.4°S	146.2°	494	0.53	20.4	3.9

**Fig. 1:**
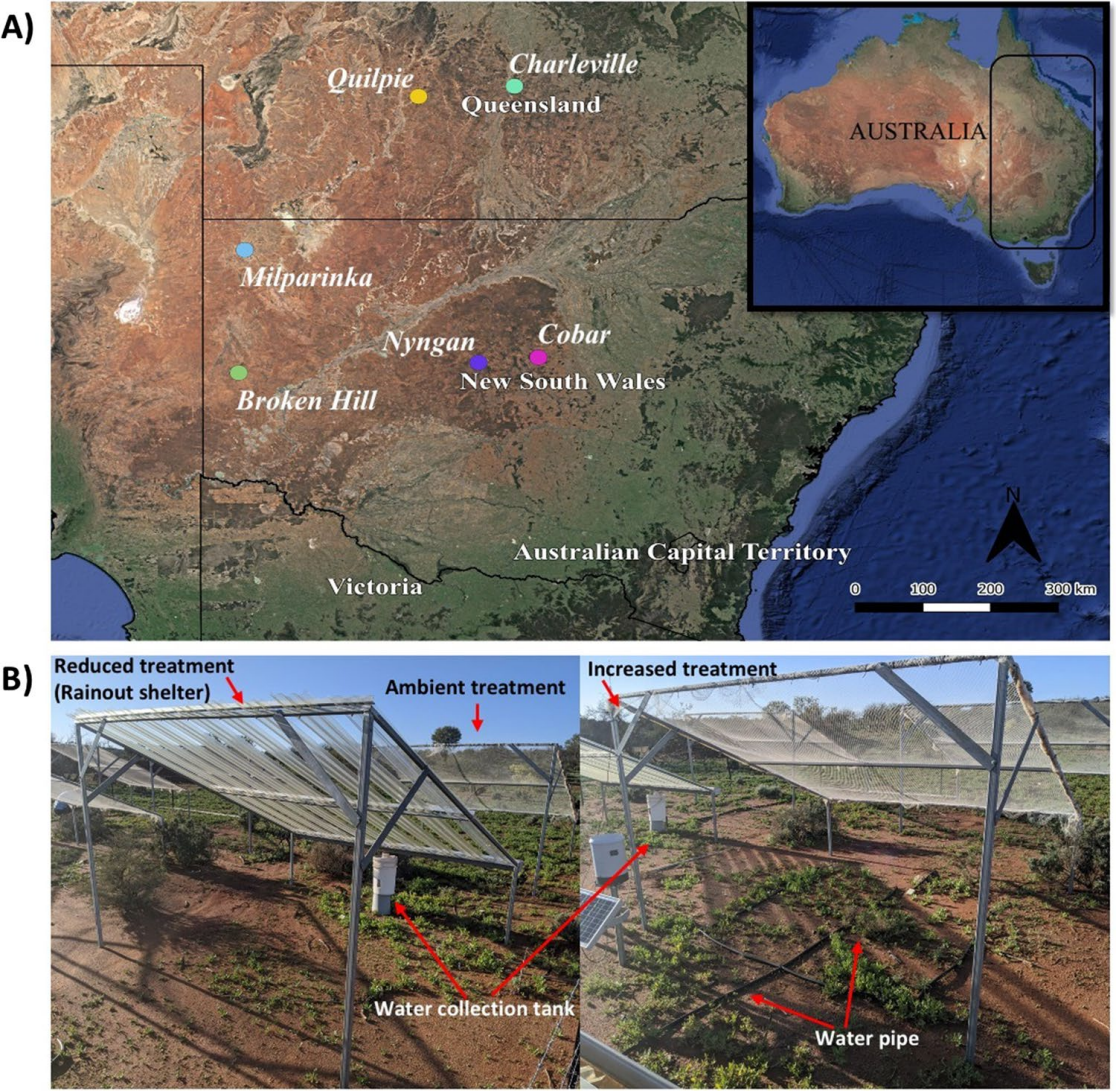
**A** Geographic location of the six dryland ecosystems including mean annual rainfall (1970–2000). Map constructed using ArcGIS Online Map Viewer (https://www.arcgis.com). **B** Example of rainfall manipulation shelters at Nyngan

Experimental manipulations at each site consist of three different rainfall treatments, representing ambient (control), reduced (-65%) and increased (~ 65%, but lower given inefficiencies in transfer of water) rainfall with three replicates at each site. Rainout shelters (3 × 3 m) use evenly spaced slats of clear acrylic plastic (allowing some rainfall to reach the ground) to exclude ~ 65% (reduced treatment) of ambient rainfall. The excess water from reduced rainfall plots is collected using gutters and transferred via polyethylene pipes to increased rainfall plots to increase water inputs equivalent to a ~ 65% increase relative to ambient rainfall (Fig. 1). Clear nylon bird netting was used as mock roofs for both ambient and increased treatment plots (allowing ambient rainfall to reach the ground). The minimum distance between plots within each site is 3 m and the maximum between plots is 21 m. The study sites are all registered with the international collaborative program Drought Network (Drought Net, https://wp.natsci.colostate.edu/droughtnet/), and the rainfall treatment is equivalent to a 1-in-100 year drought.

We conducted a vegetation survey and collected soil samples each spring (September to October) from 2016 to 2019. Data from 2016 were collected prior to imposing rainfall treatments and therefore should be considered baseline data. Between 2017 and 2019, the region experienced a severe and prolonged drought, leading to an average reduction of ~ 71% in MAP (Supplementary Material 1, Fig S1). At each timepoint, vegetation standing biomass was estimated using allometric relationships and calculated based on percentage cover of individual plant species within a 1.5 × 1.5 m quadrat centred within each plot [36]. Vegetation declined markedly in response to the coinciding severe prolonged drought (Supplementary Material 2, Table S3). In addition, eight soil cores (3.5 cm diameter, 10 cm deep) were collected randomly within each plot and combined to form a composite sample of bulk soil, with three replicate plots of each treatment per site resulting in a total of nine samples per site per year. Samples were kept cool during transport to Hawkesbury Institute of Environment (HIE), Western Sydney University (WSU), where they were subsampled for chemical analysis and microbial DNA extraction. Soils for the DNA extraction was stored at -20° C until further analyses.

### Soil Properties

Soil moisture content was estimated by oven drying ~ 10 g fresh soil in 105 C° for 2 days. Texture assessment (sand, silt, clay content) was assessed using hydrometers following Robertson et al. (1999). Soil pH was measured on a 1:5 soil:water slurry using a calibrated pH meter (S20 SevenEasy Mettler Toledo). Total soil C (TC) and nitrogen (TN) were determined using oxidative combustion method using a LECO CN Analyzer (TruMac, LECO Corporation, St Joseph, MI, USA). Total P was determined using a Epsilon 4 Benchtop X-ray fluorescence (XRF) spectrometer (Malvern Panalytical, Malvern, UK).

### Microbial Amplicon Sequencing

Microbial DNA was extracted from 0.5 g of defrosted soil using the PowerSoil DNA isolation Kit following the manufactures protocol (QIAGEN). The concentration and purity ratios of A260/230 nm and A260/280 nm of extracted DNA was visualized using Qubit fluorometry and Nanodrop spectrophotometry [37, 38]. The extracted DNA was then sent to the Western Sydney University Next-Generation DNA Sequencing facility where amplicon sequencing was conducted using paired-end sequencing on the Illumina MiSeq platform [39]. The 341F/805R primer set was used for 16S rDNA sequencing [40] and FITS7/ITS4 primer set was used for ITS sequencing to assess bacteria and fungi, respectively [41].

### Bioinformatics Analyses

The downstream processing was performed using QIIME2 (https://qiime2.org/) inbuilt plugins providing diversity and taxonomy composition analyses [42]. Initially, generated raw paired end sequence were de-multiplexed and low-quality regions and chimeric sequences were removed using the DADA2 plugin in QIIME2 [43]. Feature tables (Amplicon Sequence Variants, ASVs) and feature data (representative sequence) were generated as resulting data from DADA2. For the taxonomic classification, taxonomy was assigned to the ASVs with QIIME2 using a pre-trained Naive Bayes classifier [44] and compared against the SILVA 132 and UNITE (version 8.0) database for bacterial and fungal classification, respectively [45, 46]. The raw data bacterial and fungal sequences were deposited in Sequence read Archive (SRA) in National Centre for Biotechnology Information (NCBI) with accession number PRJNA887575.

### Statistical Analyses

Unless otherwise noted, statistical analyses were performed in R (R Core Team, 2020) and visualized using the “ggplot2” package (https://ggplot2.tidyverse.org). ASV abundance tables were randomly rarefied to an even sequencing depth of 7,400 and 20,051 for bacteria and fungi, respectively, prior to calculating alpha and beta diversity (community composition) matrices. To support the rationale for the rarefication process, a comparison model between rarefied and observed (non-rarefied) data was constructed (Supplementary 1, Fig S2). Microbial alpha-diversity was quantified using the Shannon index [47] and Chao1 richness [48]. Differences in community composition were visualized using Principal Coordinate Analysis (PCoA) using Bray–Curtis distances in the Phyloseq R package [49]. We tested for differences in microbial community diversity and composition across sites, years, and rainfall treatments including interactions among these. Additionally, distance-based redundancy analysis (dbRDA) using the Bray–Curtis distance matrix was performed to test the significance and importance of the environmental variables for community composition. These analyses were performed using the capscale function of the vegan package [50]. We analysed the statistical significance of alpha-diversity differences between sites, rainfall treatments, years and their interaction using a linear mixed effect model (ANOVA) for both bacteria and fungi. Similarly, for community composition we used Permutational ANOVA (PERMANOVA) methods to test the statistical significance using Adonis function in vegan package in R [49].

Microbiome-Analyst software (https://www.microbiomeanalyst.ca) was used to perform differential abundance (DA) analysis [51]. The data was filtered at 10% prevalence and cumulative sum scaling (CSS) was performed to identify and remove features that are unlikely to be useful when modelling the data [52]. Microbial ASVs (taxa) with significant differential abundances across year and rainfall treatments were determined using RNASeq (DESeq2) methods [53]. DESeq2 analyses were conducted using raw count data for all the ASVs. To infer results at different taxonomic levels, ASVs were grouped into taxonomic categories (e.g., phylum, class, etc.), and the differential abundance results for each category were aggregated. DESeq2 algorithm was applied to identify differential responding taxa to rainfall treatments and years for each site [53]. All items with adjusted p-values < 0.05 were considered significant.

### Co-occurrence Network Analysis

**Fig. 2:**
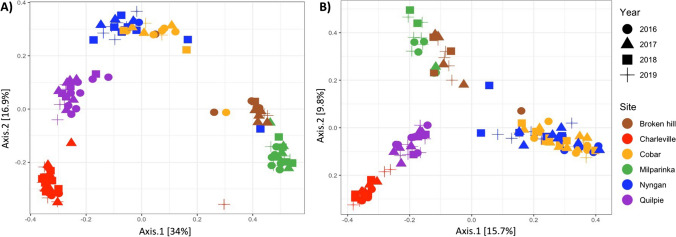
Principal Coordinate Analysis (PCoA) based on the Bray–Curtis distance measure showing the dissimilarity of bacterial (**A**) and fungal (**B**) communities across sites through time

Microbial co-occurrence networks were constructed using the molecular ecological network analyses pipeline (MENAP) (http://ieg2.ou.edu/MENA/main.cgi) with default parameters [54, 55]. Networks were constructed separately for arid sites (Milparinka, Broken Hill), semi-arid sites in QLD (Charleville, Quilpie) and NSW (Cobar, Nyngan) given clear clustering of these sites as observed in the PCoA plot (Fig. 2). Only ASVs detected in more than half of all samples of each network were kept for network construction. Each network was separated into modules by the fast-greedy modularity optimization. A modularity value measures the integrity of networks and is a fundamental characteristic of biological networks [55]. Each module in the networks represent species with similar ecological niches and functions [54, 56]. Each node in a module signifies an ASV and each edge signifies a significant (*p* < 0.05) pairwise association calculated based on Pearson correlation coefficient. The combined score of high mean degree, high closeness centrality and low betweenness centrality was used as a threshold for defining keystone taxa in microbial communities [57]. The network were visualized using Cytoscape 3.9 [58].

## Results

### Microbial Community Composition and Diversity

A total of 2,072,167 and 4,754,739 ribosomal sequences were generated for 16S (bacteria) and ITS (fungi) regions resulting in ∼1400–33100 and ∼2000–55300 sequences for each sample, respectively. Overall, bacterial communities were dominated by common phyla, including Actinobacteria (36%), Chloroflexi (26%), Proteobacteria (21%), Acidobacteria (7%), Gemmatimonadates (4%), Cyanobacteria (3%) and Planctomycetes (3%). The most dominant fungal phyla were Ascomycota (61%), Basidiomycota (19%), Chytridiomycota (4%) and Mortierellomycota (2%). The observed microbial community composition is similar to that reported for dryland soil globally (Maestre et al*.*, [10, 28] but show greater dominance of Chloroflexi (26% vs. 6.1%) and lower relative abundance of Acidobacteria (7% vs. 18%).

Principal Coordinate Analysis (PCoA) was employed to explore variation in microbial communities across sites and samples. Most of the variation between datasets was explained by site (*p* < 0.001), with both bacterial and fungal communities showing clear clustering of arid sites (Broken Hill and Milparinka), NSW semi-arid sites (Cobar and Nyngan) and QLD semi-arid sites (Charleville and Quilpie; Fig. 2). PERMANOVA analyses across the whole dataset found a significant effect of year for both bacteria and fungi (*p* < 0.01 and *p* < 0.003, respectively), but no main effect was observed for rainfall treatment for either group (Table 2). A significant interaction was observed between site and year (*p* < 0.001), and between site and rainfall treatment (*p* < 0.05), for fungal community composition, while no interaction effects were observed for bacterial community composition (Table 2).

**Table 2:** Results of the permutated multivariate ANOVA (PERMANOVA) of microbial composition (Bray–Curtis distance) with the effect of site, rainfall, year and their interactions

Bacteria					Fungi			
Factors	F-value	R square	*p*-value		F value	R square	p-value	
Site	33.218	0.581	0.001	***	13.324	0.344	0.001	***
Year	1.933	0.02	0.013	*	1.668	0.025	0.003	**
Rainfall	1.273	0.008	0.225		1.277	0.013	0.104	
Site:Year	1.254	0.065	0.067		1.378	0.109	0.001	***
Site:Rainfall	1.31	0.045	0.065		1.209	0.062	0.025	*
Year:Rainfall	0.84	0.017	0.768		0.93	0.028	0.729	
Site:Year:Rainfall	0.946	0.059	0.66		0.939	0.106	0.797	

*Significance codes: *** =  ≤ 0.001; ** =  < 0.01; * = 0.05;. =  < 0.10

To test if there were differences in microbial alpha-diversity between sites and due to rainfall and year treatments, Shannon’s and Chao1 diversity indices were calculated. The bacterial and fungal diversity was significantly influenced by site, but no rainfall or year effect were observed (Supplementary Material 1, Table S2).

**Fig. 3:**
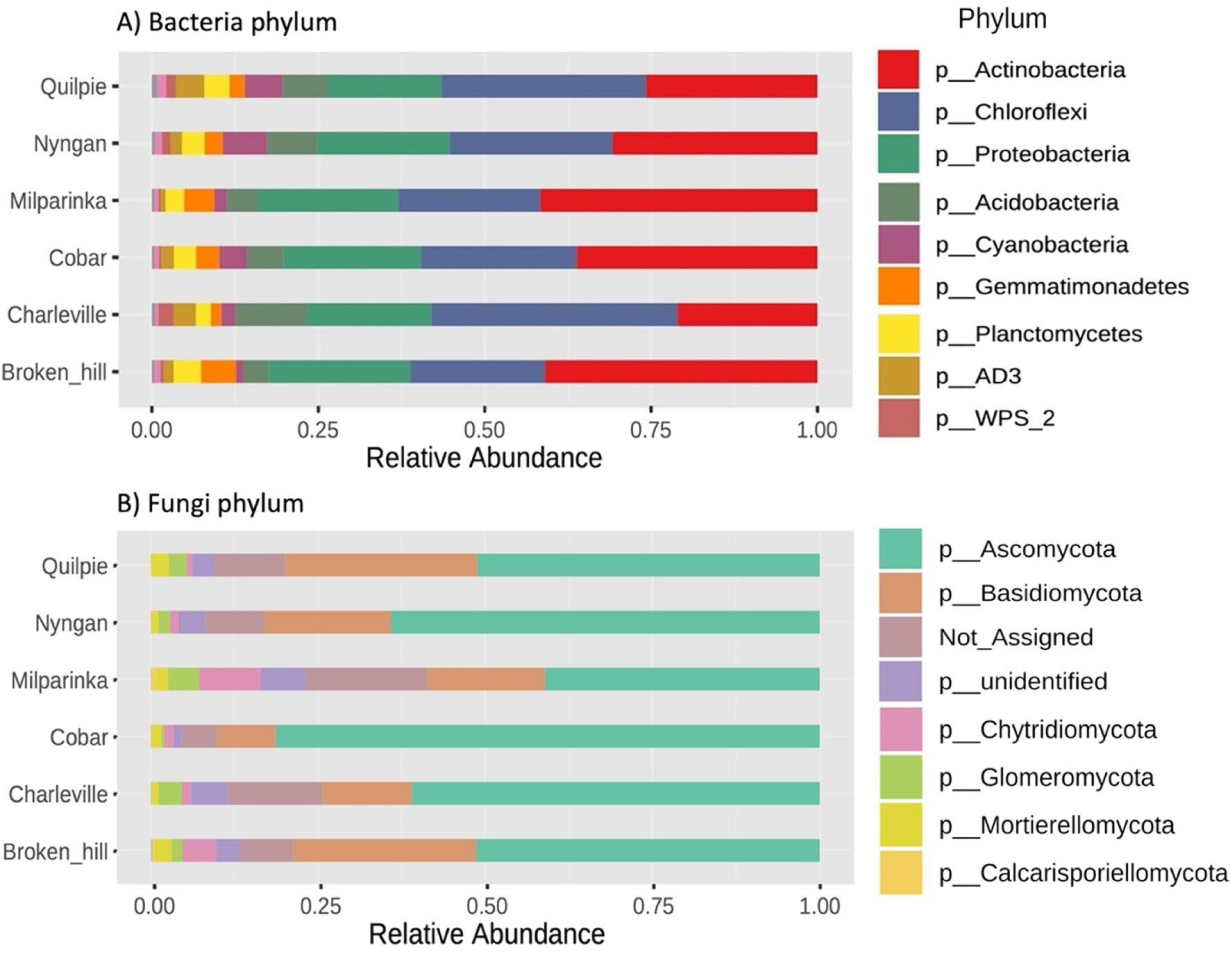
Cumulative relative abundance (%) at phylum level at each site for (**A**) Bacteria and (**B**) Fungi

To identify the microbial taxa that account for site differences, sequences were classified at phylum level. At most sites, the relative abundance of bacteria was dominated by Actinobacteria, Chloroflexi, Proteobacteria, Acidobacteria while fungi were dominated by Ascomycota, Basidiomycota and Chytridiomycota (Fig. 3). Interestingly, Chloroflexi is highly dominant at semi-arid sites while Actinobacteria were dominant at the arid sites. In terms of the fungal community, Ascomycota was more abundant at semi-arid sites while Basidiomycota were relatively more abundant at arid sites.

### Relationships between microbial communities and environmental variables

**Fig. 4:**
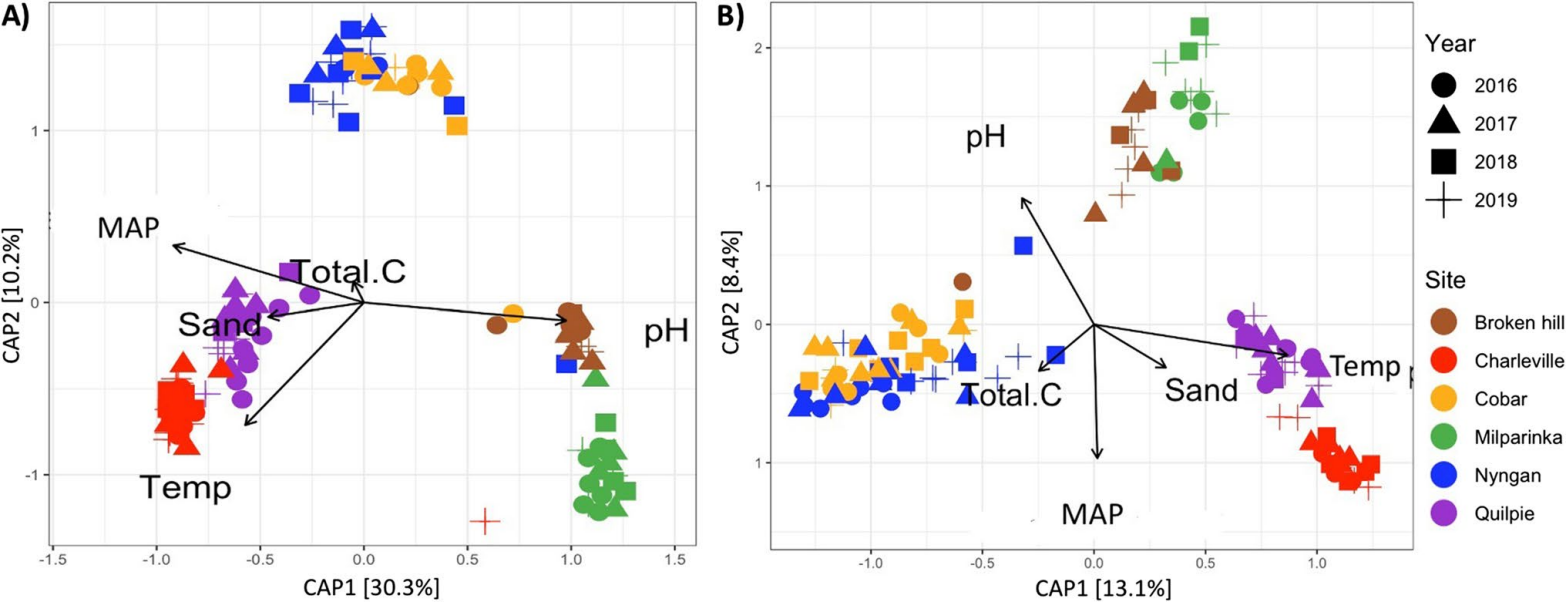
Distance-based redundancy analysis (db-RDA) biplot for Bacteria (**A**) and Fungi (**B**) including only environmental parameters that explained a significant amount of variability in microbial community structure (arrows). The direction of the arrow indicates the direction of maximum change of that variable, whereas the length of the arrow is proportional to the magnitude of change. MAP = Mean Annual Precipitation, Total.C = total soil C, and Temp = Mean Annual Temperature (MAT)

We used distance-based redundancy analysis (dbRDA) to assess relationships among microbial community composition and environmental variables across all years and sites (Fig. 4). The statistical significance of environmental variables with microbial community was tested using PERMANOVA (*p* < 0.05). Both bacterial and fungal communities were strongly related to soil pH (*p* < 0.001), soil C content (TC; bacteria = *p* < 0.005; fungi = *p* < 0.01), mean annual temperature (Temp) and rainfall (MAP) (*p* < 0.001), sand content (*p* < 0.001) associated with differences in these properties among sites, with higher soil pH and lower rainfall in the arid sites and the semi-arid sites differing mostly due to temperature.

### Differential Abundance in response to year and rainfall treatments

Differential relative abundance (DA) analyses were used to identify bacterial and fungal taxa that differed among years as the drought progressed. The DESeq2 algorithm (adjusted *p*-value < 0.05, DeSeq2) [53], originally developed for differential transcript abundance but suitable for relative count data, was used to identify indicator taxa at both high (phylum) and low (genus) taxonomic ranks. Semi-arid QLD showed the highest number of significant bacterial taxa responding to year with a total of 21 in Quilpie and 12 in Charleville. In comparison, semi-arid NSW had 14 and 5 bacterial taxa showed a significant response in Nyngan and Cobar, respectively. The arid sites had the least overrepresentations identified with both having only two significant taxa. Most of the significant taxa belonged to the phyla Acidobacteria, TM7, Gemmatimonadetes, Plactomycetes, Actinobacteria and the sub-phylum Alpha-proteobacteria. The year comparison shows that 34 taxa belonging to Acidobacteria, TM7 and Gemmatimonadetes became relatively less abundant from 2016 to 2019. On the other hand, 22 taxa including Gemmataceae, Pseudonocardiaceae, and Rhodospirillaceae, within phyla Planctomycetes, Actinobacteria and Alpha-proteobacteria, respectively, showed the opposite pattern (Supplementary Material 2, Table S1).

As for fungi, semi-arid NSW had the highest number of significant fungal taxa with a total of 71 taxa identified in Cobar and 25 in Nyngan. On the other hand, arid sites had total of 43 significant taxa identified in Broken hill and only one in Milparinka, while semi-arid QLD had a total of 34 in Charleville and only eight in Quilpie (Supplementary Material 2, Table S1). In total, 64 fungal taxa belonging to Ascomycota, Basidiomycota and Chytridiomycota showed reductions in relative abundance from 2016 to 2019, whereas 38 taxa such as family Pyronemataceae, Periconiaceae, mostly within phyla Ascomycota showed the opposite pattern (adjusted p-value < 0.05, DeSeq2, Supplementary Material 2, Table S1).

We also assessed whether taxa showed differential abundances among treatments to identify taxa that are sensitive to changes in rainfall. No significant DA bacterial taxa were identified in any of the treatments at any of the sites. In contrast, a few fungi were shown to be sensitive to the rainfall treatments with significant DA taxa identified at all sites. Semi-arid NSW and QLD sites had a total of 8 significant taxa identified as compared to arid sites with only five significant taxa identified (Supplementary Material 2, Table S2). Taxa belonging to phylum Basidiomycota were identified to be more sensitive to rainfall treatment followed by Ascomycota, limited to only semi-arid sites. Basidiomycota were predominantly higher in the reduced rainfall treatment while Ascomycota were in the increased rainfall treatment. However, the effect differed among taxa within phyla in response to rainfall treatment. For instance, Basidiomycota taxa Filobasidiales increased in relative abundance in reduced rainfall treatment, while the relative abundance of Agaricus was reduced. Similarly, Ascomycota taxa Onygenales increased with increased rainfall treatment while Pezizales was reduced (Supplementary Material 2, Table S2).

### Microbial network properties

**Fig. 5:**
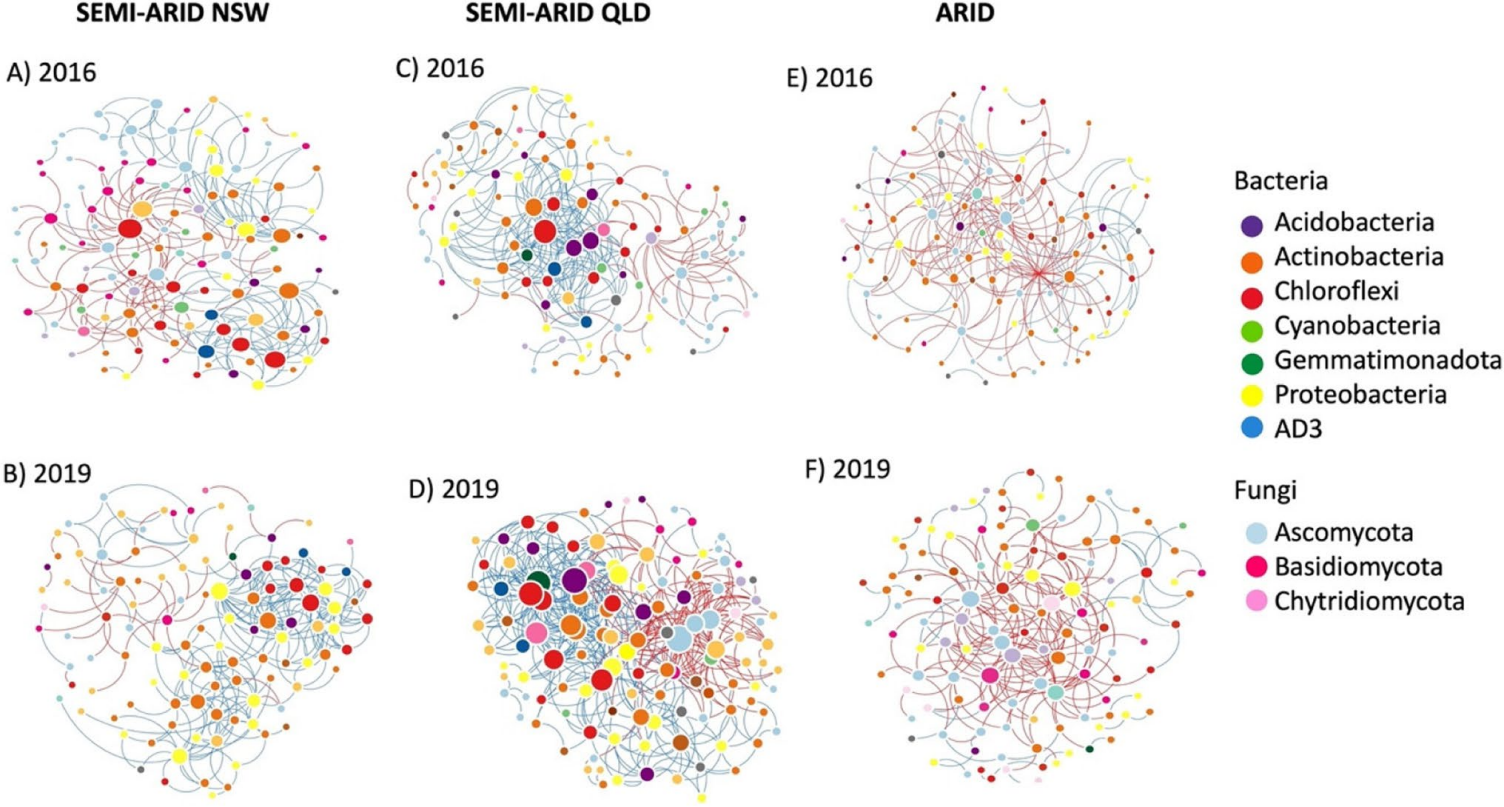
Co-occurrence networks of bacterial and fungal communities in 2016 and 2019 for semi-arid NSW (**A**,**B**), semi-arid QLD (**C**,**D**), and arid (**E**,**F**) sites. Each node represents bacterial and fungal ASVs, and each edge a significant (*P* < 0.05) pairwise Pearson association. Colours of nodes represent major taxa at phylum rank. The size of each node is proportional to the number of edges (degree). Blue and red edges indicate positive and negative interactions, respectively

Separate microbial co-occurrence networks were constructed for the sites that clustered in the PCoA (i.e., arid, semi-arid NSW, semi-arid QLD) to increase the reliability of network edges and structure and to better generate robust topological properties for network comparison. All together six networks were constructed to compare community structure in the wettest (2016) and driest (2019) years. Semi-arid soil microbial networks were noticeably more complex and had more edges and higher average clustering coefficient (avgCC) than the arid networks across both years (Fig. 5). The greater complexity of semi-arid soil explains the higher number overall network topological values as compared to arid soil (Table 3). The number of positive correlations increased from 2016 to 2019 for both the arid and semi-arid NSW sites. Interestingly, the semi-arid QLD sites had the highest number of edges, and highest number of positive links, especially in 2019, but the proportions of negative edges increased from 2016 to 2019. The avgCC, modularity and average degree (avgK) increased from 2016 to 2019 in both semi-arid and arid sites. The avgCC measure the extent of module structure present in a network, i.e., how well a node is connected with neighbouring nodes. A higher avgCC means a more complex networks, suggesting that 2019 networks were more complex than 2016. Similarly, modularity may suggest habitat heterogeneity, functional association, and phylogenetic clustering of closely related species in soil microbial communities (Olesen et al*.,* 2007).

**Table 3:** Topological metrics of co-occurrence networks in two different soil conditions of two different years. Includes average clustering coefficient (avgCC), average degree (avgK), Modularity (i.e., no. of modules). The values in parentheses following No. of edges represent the number of positive correlations

COMMUNITY	EMPIRICAL NETWORK PROPERTIES				
	No. of nodes	No. of edges	avgK	avgCC	Modularity
2016 Semi-arid NSW	125	232 (136)	3.712	0.163	0.479
2019 Semi-arid NSW	110	250 (215)	4.545	0.256	0.424
2016 Semi-arid QLD	112	269 (213)	4.804	0.218	0.645
2019 Semi-arid QLD	123	466 (326)	7.577	0.288	0.586
2016 arid	110	217 (67)	3.945	0.088	0.524
2019 arid	116	207 (138)	3.841	0.151	0.5

### Keystone microbial taxa

A total of five dominant bacterial phyla, including Chloroflexi, Proteobacteria, Actinobacteria, Acidobacteria and Planctomycetes, and three fungal phyla, Ascomycota, Basidiomycota and Chytridiomycota were detected in all networks. The phyla Chloroflexi, Actinobacteria and Ascomycota were observed to be more dominant in the networks of arid sites in both 2016 and 2019 as compared to semi-arid sites. Also, because of the important role of keystone taxa in maintaining community structure and function, the keystone taxa in the microbial communities of the generated networks were identified. The keystone taxa with the highest scores for max degree, centrality and betweenness for both semi-arid NSW and QLD in the wet year (2016) was identified as class Ktedonobacteria of phylum Chloroflexi while Thermogemmatosporaceae of phylum Chloroflexi was identified for the arid sites (Supplementary Material 1, Table S4). In the driest year (2019), the keystone taxa were identified as family Geodermatophilaceae of phylum Actinobacteria and family Botryosphaeriaceae of phylum Ascomycota in semi-arid NSW and QLD, respectively, and class Ellin 6950 of phylum Chloroflexi in the arid sites (Supplementary Material 1, Table S4).

## Discussion

This study aimed to provide insights into microbial responses to changes in rainfall related to a natural prolonged drought and with imposed long-term rainfall manipulation to better predict effects of projected climate changes on semi-arid and arid soil microbiota. Our results indicate that rainfall manipulation has limited effects on microbial communities, even after three years, with no significant shifts in community composition. However, individual taxa showed consistent responses to rainfall manipulation (especially fungi) and the coinciding prolonged drought, indicating predictable impacts of increasing aridity. Moreover, we found strong relationships among certain microbial taxa, particularly stress-tolerant taxa in this study, and how whole communities respond to aridity. Hence, stress-tolerant taxa can act as indicators for climate changes and potential shifts in dryland functional attributes.

### Microbial assemblages differ among Australian arid and semi-arid drylands but show limited responses to rainfall manipulation

Soil microorganisms, e.g. bacteria and fungi, are key players in biogeochemical cycling, but substantial differences in physiology, life history traits and functional capacity among taxa infer that taxa will show contrasting responses to environmental changes [20]. Accordingly, changes in precipitation can alter the composition and abundance of belowground microbial communities directly by changing soil water availability or indirectly by altering plants and substrate availability [27, 59]. We found strong differences in microbial community composition driven by sites and across years related to variation in rainfall among years, but the rainfall treatments had little impact observed only at a subset of the sites (Milparinka and Quilpie). Additionally, we observed that the bacterial and fungal communities are primarily grouped by their geographic location and their climatic conditions. This is in line with earlier findings where rainfall treatment effects were obscured and with sites and location being the main driver of differences and clustering of community compositions [60, 61]. In addition, microbial alpha-diversity differed among the six sites but was not affected by rainfall treatment and did not differ between years throughout the three-year study. Frequent exposure to water limitation across all sites and the intense drought observed during our study may have resulted in microbial community acclimation to fluctuations in precipitation, which might be causing diminished microbial community responses to the precipitation manipulation [23, 62]. Indeed, extreme rainfall deficiency was observed from 2017 to 2019 over much of Australia, with 2019 comparably drier than 2017 and 2018 (Bureau of Meteorology, 2020). Such severe reductions in precipitation cause soil water scarcity that compound over time, limiting plant growth and soil microbial activity [12]. The changes in the soil microbiome under drought tend to involve changes in relative abundance, with stress-tolerant taxa becoming more dominant [63] such as observed in our study.

Both bacterial and fungal communities were heavily dominated by just two or three phyla, with distributions that varied significantly by geographic location. The sites were dominated by oligotrophic taxa such as Actinobacteria, Chloroflexi, Alpha-Proteobacteria, Ascomycota and Basidiomycota that are known to tolerate drought and may out-compete other taxa in water-stressed environments [10]. Such taxa have advanced osmoregulatory functions and a strong cell wall, thereby allowing them to resist water-limited conditions [64]. Moreover, we observed an increase in the relative abundance of stress tolerance taxa with droughted years, such as Chloroflexi and Actinobacteria, which may be an indication of increasing aridity [28]. This metabolic resilience of these groups may result in the overall lack of response of microbial diversity and community composition to rainfall treatments observed in our study. This aligns with similar observations made by Ohlmann et al. [65] that mapped the importance of dominant taxa associations on changes in microbial community composition.

The differences in microbial diversity and community composition among sites were expected given the contrasting vegetation types and environmental properties. Indeed, it is well known that soil microbial community composition and abundance coincide with differences in preferred substrates and their availability, which in turn affects the soil nutrient content and cycling [66]. Our result indicated that along with MAP and MAT, soil properties such as soil pH and soil C content, play a larger role in determining microbial community composition than rainfall treatments which conforms with previous studies [67, 68]. Similarly, Fierer et al. [69] showed that the variation in dominant microbial taxa was strongly influenced by pH either directly or indirectly by affecting soil nutrients and plant root exudates. Hence, site-level characteristics such as plant diversity, soil texture, pH, MAT, and MAP, may moderate microbial functional responses to climate change. Our study focussed on microbial community responses to altered rainfall regimes and provides limited insight into shifts in functional attributes of dryland microbiomes. More targeted studies of key functional groups, such as N-fixing bacteria, and their functional response to increasing aridity under natural conditions would provide additional insight.

### Microbial taxa show differential responses to drought

Several microbial taxa showed consistent responses to the changes in rainfall imposed by the prolonged drought and our rainfall manipulations. Differential abundance analyses revealed that several taxa were abundant in the wettest year (2016) but their relative abundance decreased through time coinciding with the water limitation (Supplementary Material 2, Table S1), suggesting precipitation as a key important factor regulating soil microbial abundance [17, 70]. Interestingly, the relative abundance of Actinobacteria, Planctomycetes, Chloroflexi, Ascomycota and Basidiomycota increased over time indicating that these taxa are relatively resistant to drought stress. Gram-negative bacteria play important roles in soil nutrient cycling and are characterized as highly sensitive to drought stress [20]. Accordingly, we found that some Gram-negative taxa, including Acidobacteria, Cyanobacteria and Gemmatimonadetes, decreased in abundance with progressing natural drought. In contrast, the Gram-negative phylum Planctomycetes (especially order Gemmatales) along with Chloroflexi and Alpha-proteobacteria (especially order Rhizobiales) appeared drought resistant and increased in relative abundance under drought stress. Similarly, fungal taxa within phyla Ascomycota, especially the families Periconiaceae and Pyrometaceae of order Pleosporales and Pezizales, respectively, were found to increase in relative abundance. Most of the identified groups were plant symbionts, such as Rhizobiales, dark septate endophytes (Pleosporales) and ectomycorrhizal fungi (Pezizales), suggesting that with drought stress few niches are available for fungi and bacteria, and that plant roots constitute a major proportion of available plant habitat in these circumstances [71, 72]. Moreover, root-associated fungi like Pleosporales, are prevalent in arid soil and are able to withstand stress imposed by severe drought [72]. These groups are generally considered to be oligotrophic, slow-growing and able to survive in nutrient-poor and stressful environment [28, 69, 73]. Overall, these oligotrophic taxa play a vital role in decomposing organic matter, releasing essential nutrients back into the ecosystem. This recycling of nutrients ensures the availability of vital elements for other organisms, sustaining the overall functioning of the ecosystem [7, 8, 28], Zheng et al*.*, 2021).

In addition, several fungal taxa, including orders Filobasidiales of phylum Basidiomycota and order Onygenales and Pezizales of phylum Ascomycota, responded to rainfall manipulations suggesting that these taxa are sensitive to changes in rainfall. The Onygenales are generally soil-borne pathogenic fungi that are well adapted to extreme conditions and exhibit a competitive advantage as generalists under wet conditions [74]. This may explain their observed higher relative abundance in the increased rainfall treatment. This finding highlights the potential threat of changing rainfall patterns, as it could promote the proliferation of these pathogenic fungi, leading to an increased risk of human diseases. On the other hand, the higher abundance of saprotrophs like Filobasidiales in the reduced rainfall treatment can be attributed to their ability to thrive and persist under water-stressed conditions. These saprotrophic fungi are efficient decomposers of organic matter and can contribute to the breakdown of complex organic compounds, releasing essential nutrients back into the soil under stress conditions [73, 75]. By contrast, no bacterial taxa responded to the rainfall treatments, even though the community composition was affected by treatment at two sites (Milparinka and Quilpie). Hence, fungi appear to be more sensitive to changes in water availability in this study, contradict our hypothesis of bacteria being more sensitive to change in water availability. However, Hawkes et al. [76] reported a similar finding, where fungi were found to be more sensitive to altered precipitation. These differing responses may be due to high resilience, fast growth and high degree of physiological plasticity of bacteria as compared to fungi [11]. Thus, if their abundance is suppressed by a disturbance, they have the potential to recover quickly and return to its pre-disturbance stage [9]. In addition, we cannot dismiss the possibility that the apparent insignificance of bacterial taxa might be attributed to the DESeq2 analysis, wherein the adjusted p-value may not be sensitive enough to detect significant changes within these taxa. Nevertheless, the overall decreased in relative abundance of several bacterial and fungal taxa implies stronger impact of prolonged drought and higher vulnerability in dryland soil.

### Prolonged drought influences microbial network structure

We observed distinct differences in microbial community composition and structure between arid and semi-arid sites, with semi-arid ecosystems being more sensitive to prolonged drought as hypothesized by [17]. These differences are also reflected in our correlation-based network structure comparing arid and semi-arid dryland ecosystems in 2016 and 2019 (Fig. 3 and Table 3). The number of positive correlations (links) increased from 2016 to 2019 following the drought for both semi-arid NSW and arid microbial networks, with more complex networks observed in semi-arid as compared to arid ecosystems. This outcome can arise when highly competitive taxa involved in numerous antagonistic interspecific interactions are gradually replaced by slow-growing, stress-tolerant species (such as oligotrophic microbes) as stress increases [71, 77], as observed in our study. However, the proportion of negative interactions increased in semi-arid QLD, suggesting microbial interkingdom interactions and favouring resource competitive mechanisms between phylogenetically distant microorganisms [78]. This finding coincides with the ‘stress gradient hypothesis” where harsh environments such as drought can strongly favour microbial synergism, but will also depend on the level of underlying resource competition (Piccardi, Vessman and Mitri, 2019). Furthermore, persistent stress, such as long-term drought stress in our study, can promote network properties with characteristics of unstable communities [71]. This finding helps explain the increased variation in the relative abundance of microbial communities in response to temporal variability. Moreover, most taxa belonging to phylum Chloroflexi were identified as keystone taxa along with phylum Actinobacteria and Ascomycota. The identified keystone taxa have been shown to be associated with litter decomposition and removing these taxa affected the overall microbial community structure (Zheng et al*.*, 2021). Therefore, they may play critical roles in maintaining the structure and function of dryland soil microbial communities in future climates. However, correlation network analyses are only a simplistic statistical correlation representation of a complex system and can yield spurious results [79]. Nevertheless, ecological network study is still useful for offering insights into the topological properties of microbial community members and are regarded as a valuable tool to identify species associations within a community (Proulx, Promislow and Phillips, 2005; [80].

## Conclusion

Our results suggest that spatiotemporal variation in aridity plays a major role in shaping dryland microbial communities although responses are moderated by their geographic location and associated environmental factors. Such variation can be primarily driven by differences in soil abiotic factors, such as pH and soil C content with changes in rainfall amount limited to fungi. Specifically, drought stress increases the relative dominance of stress-tolerant microbes (i.e., oligotrophic taxa) mostly belonging to phylum Actinobacteria, Planctomycetes, Chloroflexi, Ascomycota, Basidiomycota, which given their life history characteristics will have cascading effects on ecosystem functions. Moreover, our results highlight that the stress-tolerant keystone taxa such as Chloroflexi, Actinobacteria and Ascomycota may influence community composition through its effects on other subsidiary taxa and thus, can be used to guide future enrichment efforts in drylands. These findings provide an opportunity to better understand and predict microbial responses to increases in rainfall variability and the associated effects on the functioning of semi-arid and arid ecosystems. Consequently, comprehending the long-term effects of drought on microbiome stability holds significant implications for species distribution modelling, ecosystem services, and agricultural practices in these environments.

### Supplementary Information

Below is the link to the electronic supplementary material.

Supplementary file1 (XLS 134 KB)

Supplementary file2 (DOCX 3515 KB)
