# Supplementary material for: Severe Prolonged Drought Favours Stress-Tolerant Microbes in Australian Drylands
Source: Microb Ecol. 2023 Oct 25;86(4):3097–110. doi: 10.1007/s00248-023-02303-w (PMC10640424; doi:10.1007/s00248-023-02303-w)
Supplement: Supplementary file 2 — Supplementary file2 (DOCX 3515 KB) [file 248_2023_2303_MOESM2_ESM.docx]

**Supplementary Material 1**

*Table S1. Description of the sites, including dominant vegetation species and soil properties.*

| Site | Dominant species at the site | Soil description | Texture | | | pH |
| --- | --- | --- | --- | --- | --- | --- |
|  |  |  | Sand (%) | Silt (%) | Clay (%) |  |
| Broken Hill | Maireana pyramidata | Brown calcareous sand | 62 | 11.89 | 26.1 | 7.3 |
| Cobar | Eordium crinitum, Helipterum species, Medicago species, Ptilotus species, and Austrostipa scabra | Lowland floodplain red soil, clay with minimal gravel | 68.8 | 7.56 | 23.7 | 6.5 |
| Nyngan | Calotis lappulacea, Erodium crinitum, Austrostipa scabra, Helipterum species, and Medicago species | Upland floodplain red soil, clay with some gravel | 81.6 | 4.22 | 14.2 | 6 |
| Milparinka | Astreblea lappeacea, A. pectinata, and Abutilon halophilum | Grey ‘cracking’ clay | 72.9 | 11.4 | 15.6 | 8 |
| Quilpie | Eragrostis setifolia, Sclerolaena species, and Abutilon halophilum | Hard red clay, gravelly | 80.7 | 7.11 | 12.2 | 5.4 |
| Charleville | Eremophila gilesii (native invasive woody scrub) | Red sandy, porous | 58.5 | 10.44 | 31.1 | 4.9 |

**A)**

**B)**

*Fig S1. The boxplot illustrates the Mean Annual Rainfall (mm) for each site from the year 2016 to 2019 (A) and the line graph shows the amount of rainfall scaled to the three treatments, i.e., increased (+50%), reduced (-65%) and ambient rainfall (B). During the period from 2017 to 2019, a significant drought was observed, with approximately 71% reduction in rainfall compared to the baseline year i.e., 2016.*

1. **B)**

*Fig S2. The rarefaction plot compares (A) non-rarefied (Observed) and (B) rarefied fungal richness of two sites (Broken hill and Milparinka). The rarefied plot demonstrates a more even sequencing depth compared to the un-rarefied plot****.***

A)

B)

C)

D)

*Fig S3. Bacterial (A) (B) and fungal (C) (D) chao1 richness and Shannon diversity across sites and treatments. Asterisks denote significant correlation (**p < 0.01, *p < 0.05) and ns (not significant)*

*Table S2: Result of linear mixed effect models (ANOVA) for bacteria and fungi testing for differences in diversity and richness among sites, year, and rainfall treatments, including their interactions. *Signif. codes: 0 ‘***’ 0.001 ‘**’ 0.01 ‘*’ 0.05 ‘.’ 0.1 ‘ ’ 1*

| **Effect** | **Bacteria** | | | | | |
| --- | --- | --- | --- | --- | --- | --- |
|  | **Shannon diversity** | | | **Chao1** | | |
|  | F-value | p-value | Significance codes | F-value | p-value | Significance codes |
| **Site** | 3.112 | 0.01 | * | 8.133 | 6.09E-07 | *** |
| **Year** | 1.722 | 0.19 | ns | 0.259 | 0.611 | ns |
| **Rainfall** | 0.443 | 0.642 | ns | 1.762 | 0.174 | ns |
| **Site:Year** | 3.766 | 0.002 | ** | 1.603 | 0.161 | ns |
| **Site:Rainfall** | 0.719 | 0.705 | ns | 1.735 | 0.075 | . |
| **Year:Rainfall** | 0.0243 | 0.975 | ns | 0.674 | 0.0509 | ns |
| **Site:Year:Rainfall** | 1.141 | 0.334 | ns | 0.759 | 0.667 | ns |
|  | **Fungi** | | | | | |
|  | **Shannon diversity** | | | **Chao1** | | |
|  | F-value | p-value | Significance codes | F-value | p-value | Significance codes |
| **Site** | 3.041 | 0.011 | * | 10.027 | 1.76E-08 | *** |
| **Year** | 1.53 | 0.217 | ns | 0.969 | 0.969 | ns |
| **Rainfall** | 0.393 | 0.675 | ns | 0.521 | 0.594 | ns |
| **Site:Year** | 3.803 | 0.002 | ** | 2.093 | 0.068 | . |
| **Site:Rainfall** | 0.761 | 0.665 | ns | 1.005 | 0.44 | ns |
| **Year:Rainfall** | 0.011 | 0.988 | ns | 0.414 | 0.661 | ns |
| **Site:Year:Rainfall** | 1.228 | 0.275 | ns | 0.624 | 0.792 | ns |

*Table S3. PERMANOVA result testing for differences in microbial composition (Bray-Curtis distance indices) between rainfall treatments and year for each site.*

| **Bacteria** | | | | | **Fungi** | | | |  |
| --- | --- | --- | --- | --- | --- | --- | --- | --- | --- |
| **Site** | **Factors** | **F-value** | **R square** | **p-value** |  | **F value** | **R square** | **p-value** |  |
| **Broken Hill** | **Rainfall** | **0.964** | **0.056** | **0.478** |  | **1.159** | **0.065** | **0.206** |  |
|  | **Year** | **1.654** | **0.138** | **0.007** | ****** | **2.056** | **0.161** | **0.001** | ******* |
| **Milparinka** | **Rainfall** | **1.671** | **0.092** | **0.039** | ***** | **1.509** | **0.083** | **0.025** | ****** |
|  | **Year** | **1.451** | **0.119** | **0.078** |  | **1.288** | **0.107** | **0.069** |  |
| **Cobar** | **Rainfall** | **1.415** | **0.078** | **0.088** |  | **0.605** | **0.035** | **0.663** |  |
|  | **Year** | **1.353** | **0.112** | **0.092** |  | **1.874** | **0.149** | **0.004** | ****** |
| **Nyngan** | **Rainfall** | **0.648** | **0.037** | **0.936** |  | **1.091** | **0.062** | **0.299** |  |
|  | **Year** | **2.052** | **0.161** | **0.002** | ****** | **1.874** | **0.149** | **0.001** | ******* |
| **Charleville** | **Rainfall** | **0.713** | **0.041** | **0.717** |  | **1.049** | **0.059** | **0.37** |  |
|  | **Year** | **2.052** | **0.161** | **0.002** | ****** | **1.919** | **0.152** | **0.001** | ******* |
| **Quilpie** | **Rainfall** | **2.117** | **0.113** | **0.002** | ****** | **1.259** | **0.071** | **0.106** |  |
|  | **Year** | **2.145** | **0.167** | **0.001** | ******* | **0.122** | **0.122** | **0.007** | ****** |

*Table S4. Network topology of identified keystone taxa with phylum in bracket.*

| **Year** | **Nodes value** | **Semi-arid NSW** | **Semi-arid QLD** | **Arid** |
| --- | --- | --- | --- | --- |
|  |  | Ktedonobacteria (Chloroflexi) | Chloroflexi | Thermogemmatisporaceae (Chloroflexi) |
| **2016** | Max degree | 17 | 31 | 35 |
|  | Max betweenness | 1561.527 | 1289.14 | 2512.303 |
|  | Max stress centrality | 6634 | 6916 | 12103 |
|  |  |  |  |  |
|  |  | Geodermatophilaceae (Actinobacteria) | Botryosphaeriaceae (Ascomycota) | Ellin6950 (Chloroflexi) |
| **2019** | Max degree | 18 | 28 | 16 |
|  | Max betweenness | 1018.349 | 1237.658 | 962.073 |
|  | Max stress centrality | 3748 | 8852 | 2748 |
